# Assessment of pleiotropic transcriptome perturbations in *Arabidopsis* engineered for indirect insect defence

**DOI:** 10.1186/1471-2229-14-170

**Published:** 2014-06-19

**Authors:** Benyamin Houshyani, Alexander R van der Krol, Raoul J Bino, Harro J Bouwmeester

**Affiliations:** 1 Wageningen University, Plant Sciences Group, Laboratory of Plant Physiology, P.O. Box 658, Wageningen 6700 AR, The Netherlands

**Keywords:** Genetic modification, Assessment, *Omics*, Transcriptome distance, Substantial equivalence, *Arabidopsis*

## Abstract

**Background:**

Molecular characterization is an essential step of risk/safety assessment of genetically modified (GM) crops. Holistic approaches for molecular characterization using *omics* platforms can be used to confirm the intended impact of the genetic engineering, but can also reveal the unintended changes at the *omics* level as a first assessment of potential risks. The potential of *omics* platforms for risk assessment of GM crops has rarely been used for this purpose because of the lack of a consensus reference and statistical methods to judge the significance or importance of the pleiotropic changes in GM plants. Here we propose a *meta data* analysis approach to the analysis of GM plants, by measuring the transcriptome distance to untransformed wild-types.

**Results:**

In the statistical analysis of the transcriptome distance between GM and wild-type plants, values are compared with naturally occurring transcriptome distances in non-GM counterparts obtained from a database. Using this approach we show that the pleiotropic effect of genes involved in indirect insect defence traits is substantially equivalent to the variation in gene expression occurring naturally in *Arabidopsis*.

**Conclusion:**

Transcriptome distance is a useful screening method to obtain insight in the pleiotropic effects of genetic modification.

## Background

According to the consensus document on the assessment of plants derived from modern biotechnology, a molecular characterization must be included to provide assessors with the possibility to predict phenotypes and risk/safety concerns [1]. To be able to do so, reliable methods and tools for characterization and risk/safety assessment are essential. To assess the risk of novel foods^2^ (such as a GM food^3^), the term “substantial equivalence” was introduced by the OECD in 1991. This concept implies that a novel food should be considered the same as and as safe as a conventional food (the safe counterpart) if it has the same characteristics and composition. Development of reliable methods and tools to analyse the equivalence thus is important from a regulatory point of view; such methods could also be used for the evaluation of GM crops, in principle making it possible that they are evaluated under the same regulatory framework as their non-GM counterparts **(**CRS Report for Congress: 2005; http://www.cnie.org/NLE/CRSreports/05jun/97-905.pdf).

The untargeted measurement techniques collectively referred to as “*omics*” (proteomics, metabolomics and transcriptomics) can be used for characterizing and evaluating the effects of transgene insertion and compositional equivalence of GM crops relative to their conventional counterparts [2-7]. These techniques should confirm the intended impact of the novel trait^4^ (has the intended change occurred) but can also reveal the unintended changes. To judge whether an unintended change is significant, however, the magnitude of the changes should be evaluated and judged against a *baseline* representing the natural variation in the trait under evaluation (proteome, metabolome and/or transcriptome) in the natural parental lines [6,8], wild relatives [9,10], populations derived from the parental lines and populations exposed to naturally occurring biotic and/or abiotic stress factors [6,7,11]. Such a comparison is not trivial, as for each sample thousands of data points are generated with each of these *omics* technologies. An example, where such statistical analysis was successfully used, is our earlier work [12] in which a method that determines the metabolic ‘hyper-plane distance’ between samples was presented.

**Figure 1:**
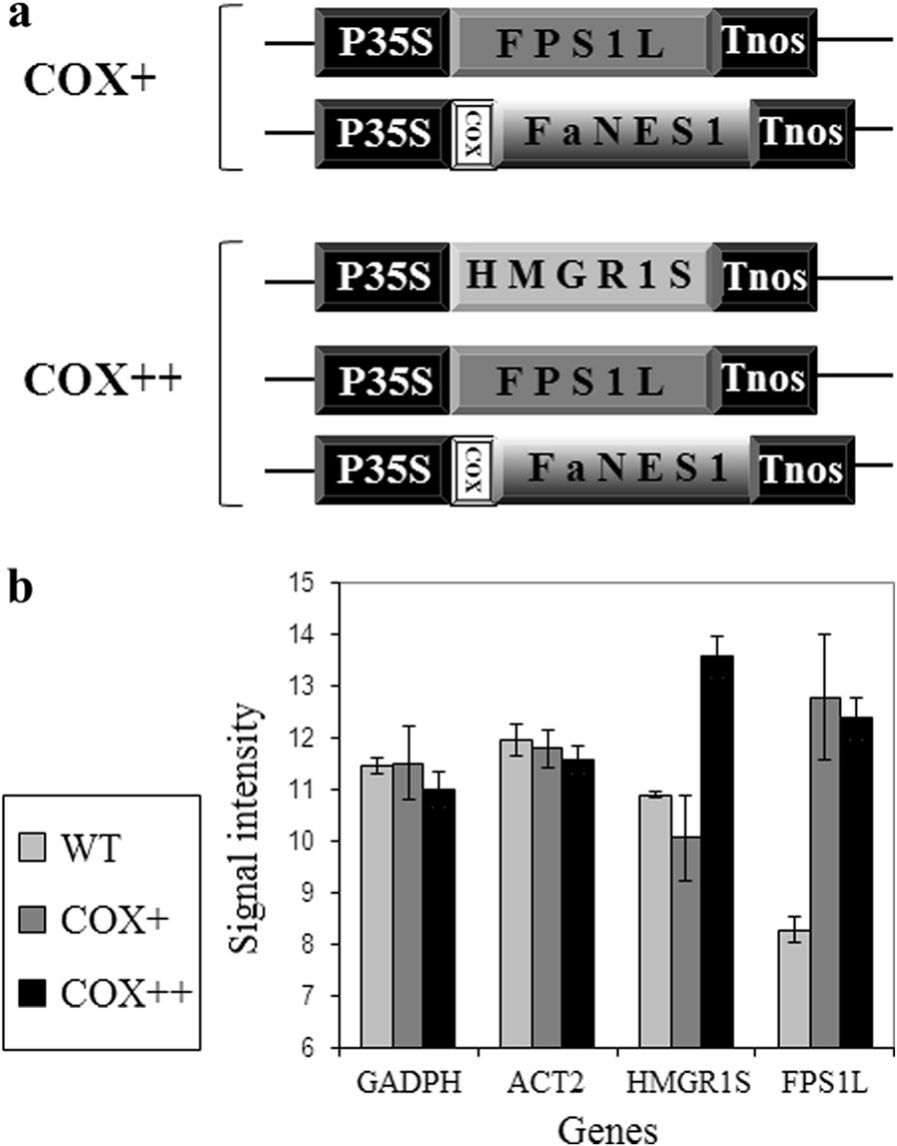
**Constructs used for transgene introduction in *Arabidopsis. *(a)** Genes stacked in COX + and COX++ lines by crossing: *HMGR1S*, short (cytosolic) isoform of 3-hydroxy-3-methylglutaryl coenzyme A reductase 1; *FPS1L*, farnesyl diphosphate synthase 1 long isoform; *FaNES1*, nerolidol synthase 1 from *Fragaria* x *ananassa*; COX, mitochondrial signal peptide; P35S, CaMV 35S promoter; Tnos, terminator of the *Agrobacterium tumefaciens* nopaline synthase. **(b)** Average signal intensities of the overexpressed (*HMGR1S* and *FPS1L*) and housekeeping (*GADPH* and *ACT2*) genes in Col-3 (WT), COX + and COX++ lines. Bars indicate the 95% confidence interval.

In the present study, we analysed the transcriptome changes in two genetically modified (GM) *Arabidopsis* lines. Both lines expressed a mitochondrial targeted nerolidol synthase gene (*COX-FaNES1*), which makes the GM plant attractive to the natural enemies (predatory mites) of spider mites [13]. On top of that, two strategies were followed to boost expression of the trait by improving substrate availability. In one line (COX+) the precursor availability was boosted by overexpression of a mitochondrial targeted farnesyl diphosphate (FPP) synthase (*FPS*) 1 long isoform (*FPS1L, At5g47770*) (Figure 1a) [14]. In the other line (COX++), precursor availability was boosted even further by overexpression of both *FPS1L* and a cytosolic truncated hydroxymethylglutaryl CoA reductase 1 short isoform (*HMGR1S, At1g76490*) (Figure 1a). *HMGR1S* encodes an enzyme catalysing a rate-limiting step in the mevalonate pathway and *FPS1L* encodes an enzyme catalysing the formation of the direct substrate for sesquiterpene synthases. Both represent pleiotropic and rate limiting enzymes [15,16]. Both lines emit the volatile compound, (*E*)-nerolidol in the headspace and are similarly efficient in attraction of the endolarval parasitoid of *Plutella xylostella* (diamondback moth), *Diadegma semiclausum *[17]. The use of different strategies to generate the same trait can help to classify potential changes in the transcriptome into changes that are specifically associated with the novel trait(s) and changes that are non-specific.

**Figure 2:**
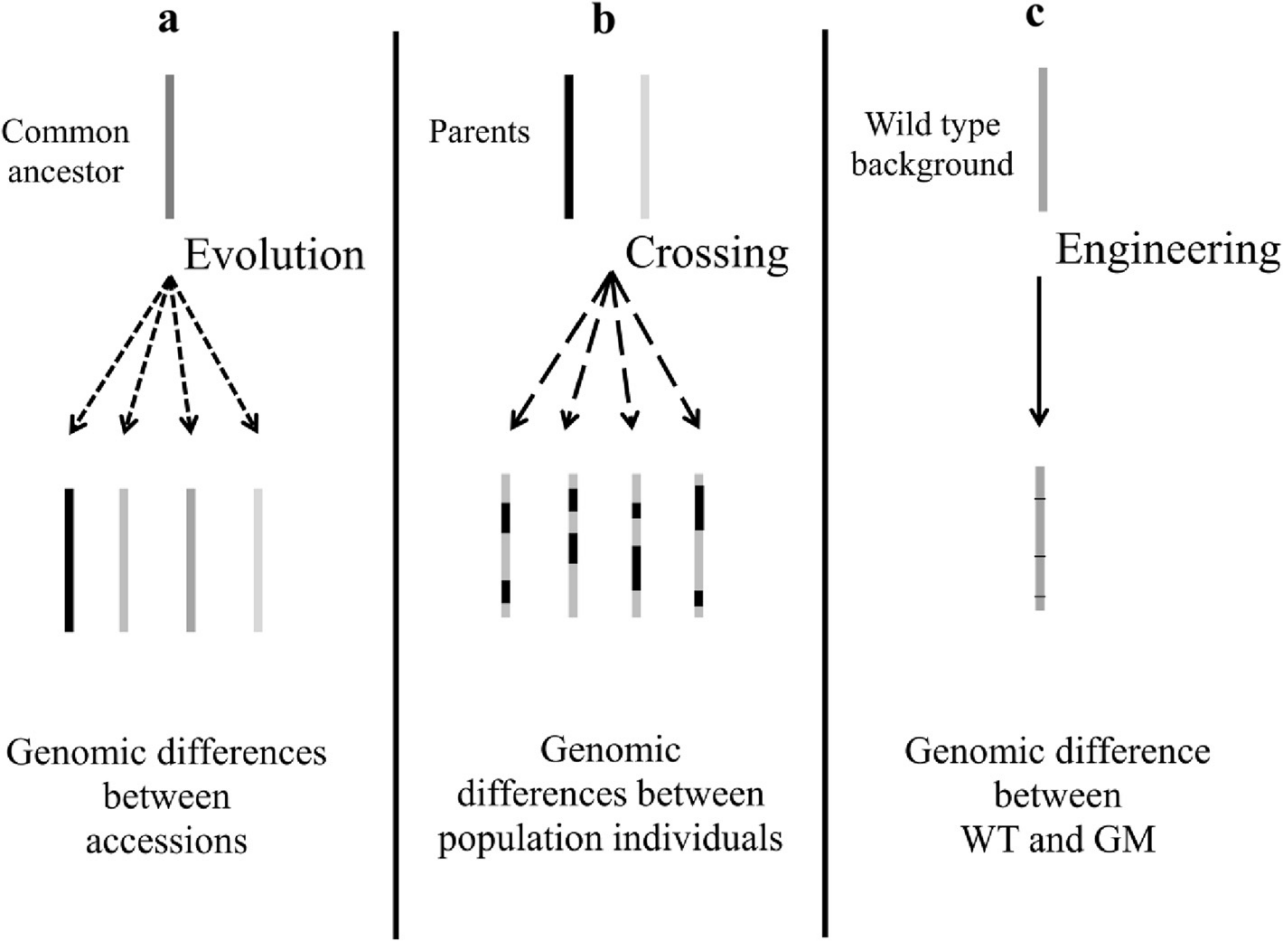
**Potential genetic sources for transcriptome variation. (a)** genomic differences between accessions. **(b)** genomic differences between population individuals. **(c)** genomic differences between a genetically modified individual and the corresponding wild type. Each bar represents the whole genome of an individual line. WT, wild type. GM, genetically modified.

The following workflow was used to analyse the transcriptome changes in *Arabidopsis*: 1- changes in the transcriptome of the transformed lines were first compared to the Col-3 (wild type) background, 2- intended and unintended changes that form the overall perturbation were analysed and 3- the substantial transcriptome equivalence of the overall perturbation was statistically evaluated by comparing the transcriptome changes with natural *Arabidopsis* transcriptome variation, the *baseline*. Hereto, we used microarray data of the transgenic lines that were generated specifically for this study and compared them with data from publicly available databases on 16 *Arabidopsis* accessions and four groups of lines derived from an *Arabidopsis* RIL population (Ler/Cvi). The transcriptomics data for the *Arabidopsis* accessions represent the genetic transcriptome variability caused by diversification of a common ancestor’s genome. This variability is achieved by natural mutations combined with local evolutionary selection pressure, resulting in diverse but supposedly balanced genome compositions of the different accessions and consequently different transcriptional profiles (Figure 2a). The RIL population represents the genetic diversity caused by mixing the Cvi and Ler genomes and hence is representative for domestication of plant species through modern breeding (Figure 2b). The recently developed statistical method to determine the metabolic ‘hyper-plane distance’ [12] was adapted to calculate a ‘*transcriptome distance*’ between groups of samples which allows comparison of the substantial transcriptome equivalence of GM lines with the *baseline*.

### Definitions

1) OECD, The Organization for Economic Co-operation and Development (OECD) is an international economic organization to stimulate economic progress and world trade. It is a forum of countries committed to democracy and the market economy, providing a platform to compare policy experiences, seek answers to common problems, identify good practices, and co-ordinate domestic and international policies of its members.

2) Novel food is a type of food that does not have a significant history of consumption or is produced by a method that has not previously been used.

3) Genetically modified (GM) foods are foods derived from genetically modified organisms. Genetically modified organisms have had specific changes introduced into their DNA by genetic engineering techniques.

4) Trait in this paper is the characteristic that is the aim of genetic modification.

## Results

Transformation of Col-3 plants used in this study was done by the insertion of 2 (in COX+) or 3 (in COX++) constructs, each containing the gene of interest (*COX-FaNes1*, *FPS1L* or *HMGR1S*) (Figure 1a) and a selection-marker gene [17]. Quantitative RT-PCR showed *de novo FaNES1* expression in the transgenic lines. *FPS1L* expression was at least 64-fold higher in COX + and COX++ and expression of *HMGR1S* was at least16-fold higher in COX++ lines than in Col-3 (the wild type) [17].

For the analysis of the transcriptome in the GM and wild type plants, RNA of Col-3, COX + and COX++ transgenic lines (each represented by 3 biological replicates) was isolated for *Arabidopsis* ATH1 GeneChip hybridization. Sampling was in a stage at which the GM plants produced a volatile blend that attracted parasitoid wasps (*Diadegma semiclausum*) [17]. *FPS1L* and *HMGR1S* are *Arabidopsis* genes and are present on the *Arabidopsis* micro-array. The average signal intensity for *FPS1L* was 22.6 fold (4.5 units) higher in COX + or COX++ than in the Col-3 and for *HMGR1S* the increase in signal intensity was 5.7 fold (2.5 units), which is similar to the quantitative RT-PCR results (Figure 1b). Microarray analysis produced expression data for 22746 probe sets (genes) that were entirely used in the rest of the analyses. Applicability and quality of microarray data was confirmed and no outlier could be detected within the biological replicates. Therefore, the existing variability across the biological replicates was accepted for the rest of analyses.

### Transcriptome changes

#### ***Quantitative changes in the transcriptome***

Comparing COX + lines with Col-3 plants, 545 probe sets (genes) were differentially expressed (2.4% of the total) of which 139 (0.6%) and 35 (0.2%) were more than two fold up- or down-regulated in COX + lines, respectively. In the COX++ versus Col-3 comparison, the number of differentially expressed genes was 485 (2.1% of total) of which 13 (0.1%) were more than two fold up- and 85 (0.4%) were more than two fold down-regulated. Only 131 genes (0.6% of total) were differentially expressed in both modifications of which 47 (0.2%) were more than two fold up- or down-regulated in at least one of the modifications. A closer look at the list of 47 genes and their pattern of change revealed that only four genes have the same pattern of change, one up-regulated (*At5g47770*, *FPS1*) and three down-regulated (*At4g29020*, *At3g30720*, *At3g50360*) in both COX + and COX++ lines while the other genes showed a reverse pattern between the two transgenic lines. Combined, these results indicate a negligible overlap (or a remarkable difference) between the two transgenic strategies with respect to their impact on the transcriptome of Col-3.

**Figure 3:**
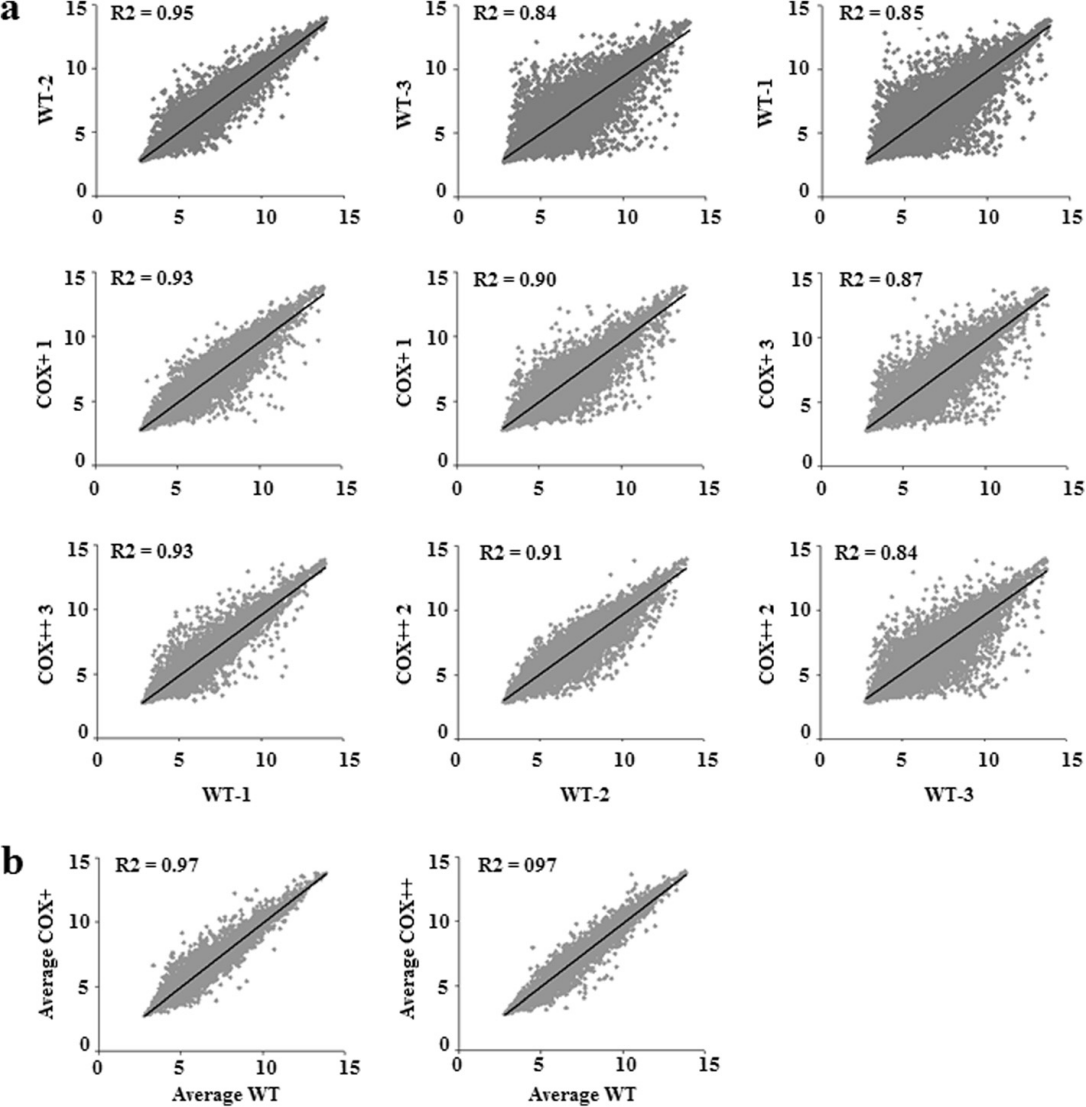
**XY scatter plots of transcriptome data.** The correlation constant (R^2^) represents the variability of the global gene expression profile of two samples or groups. A small R^2^ indicates large variation, a large R^2^ indicates small variation. **(a)**. XY scatter plots of the Col-3 replicates (WT) with each other (1st row) and with a replicate of COX + and COX++ with the smallest R^2^ value (2nd and 3rd row, respectively). **(b)**. XY scatter plot for the averages of the groups.

XY scatter plots allow visualization and comparison of the global gene expression variation across Col-3 and the transgenic lines. First the variability between Col-3 replicates was used to establish the *baseline* variation in these XY scatter plots (Figure 3a, 1st row). XY scatter plot of an individual Col-3 replicate and a transgenic line of COX + and COX++ group with the smallest R^2^ (therefore the highest variability) are shown in Figure 3a 2nd and 3rd row, respectively. Comparison of the Col-3 scatter plots (*baseline*) with that of transgenic lines showed no obvious influence of the genetic modification on the global gene expression pattern as inter-Col-3 correlations have an equal or smaller R^2^ (thus larger variation) than the correlation of transgenic lines with Col-3 (Figure 3a). XY scatter plots for the group averages show that the averaged Col-3 gene expression values are globally a reliable predictor for the expression of most of the genes in both transgenic groups with an R^2^ value of 0.97 for both the Col-3 *Vs.* COX + and COX++ scatter plots (Figure 3b).

#### ***Gene Set Enrichment analysis of transcriptional changes***

Gene Set Enrichment analysis (GSEA) [18,19] was used to determine whether any *a priori* defined sets of genes (555 sets) [20] are differentially expressed between Col-3 and COX + and COX++ transgenic lines. Instead of analysing the correlation of a single gene with the new biological state (GM), GSEA derives its power from looking at the effect of genetic modification in sets of genes that share a common biological function, cellular localization, chromosomal location or regulation.

None of the gene sets showed a significant change in the transgenic lines compared with Col-3 in GSEA. The absence of a significant difference shows there is negligible variation caused by the introduction of the transgenes compared with other sources of variation. GSEA also resulted in a list of genes ranked by their correlation with *FPS1L* expression. The heat map (Additional file 1: Figure S1) shows that 17 genes have more than 90% correlation with the expression level of *FPS1L* (six positively and 11 negatively). This list also includes the three genes that were more than two fold down-regulated in both transgenic lines, *At4g29020* (encoding an endomembrane glycine-rich protein), *At3g30720* (encoding qua-quine starch, a cytosolic protein) *and At3g50360* (encoding centrin2, the plasma membrane calcium binding protein) none of which we can currently link biologically to either *FaNES1* expression or *FPS1L* overexpression.

#### ***Analysis of functional categories in transcriptional changes***

Using over-representation analysis (ORA), the enrichment of functional categories in the set of significantly up- or down-regulated genes was analysed. A set comprising all probe set IDs of the *Arabidopsis* ATH1 GeneChip was used as a reference for statistical evaluation.

Ignoring the fold change criteria, 318, 154 and 29 genes were significantly up-regulated in COX+, COX++ and both lines, respectively, compared with the wild type Col-3 (ANOVA in conjunction with Tukey for pair-wise comparison, α = 0.05 as the threshold for difference significance). The first two sets significantly represented 198 and 16 categories in COX + and COX++ lines compared with the reference set, respectively (over-representation analysis [ORA] with false discovery rate [FDR], 0.05). No functional over-represented category was present in both transgenic lines confirming the absence of any similarity between the transcriptome changes in the COX + and COX++ lines. Ignoring fold change in down-regulation, 227, 330 and 34 genes were significantly down-regulated in COX+, COX++ and both lines, respectively, compared with the wild type Col-3 (ANOVA in conjunction with Tukey, α = 0.05). The first two sets significantly represented 59 and 242 categories in COX + and COX++ compared with the reference set, respectively (over-representation analysis [ORA] with false discovery rate [FDR], 0.05). Only the “protein metabolic process” category was significantly represented in both lines but under-represented in COX + and over-represented in COX++.

In order to narrow down our search for functional categories that could be specifically related to the molecular mechanism behind the novel trait, over-representation analysis (ORA) was done on the commonly up- or down-regulated genes in both COX + and COX++ lines. Among the 29 up-regulated and the 34 down-regulated genes in both COX + and COX++ lines, no specific functional category was over- or under-represented (over-representation analysis [ORA] with false discovery rate [FDR], 0.05). This indicates that common transcriptome changes in COX + and COX++ lines are rare and that the novel trait does not induce defined changes in certain biological categories.

#### ***Multivariate analysis of GM transcriptome data***

**Figure 4:**
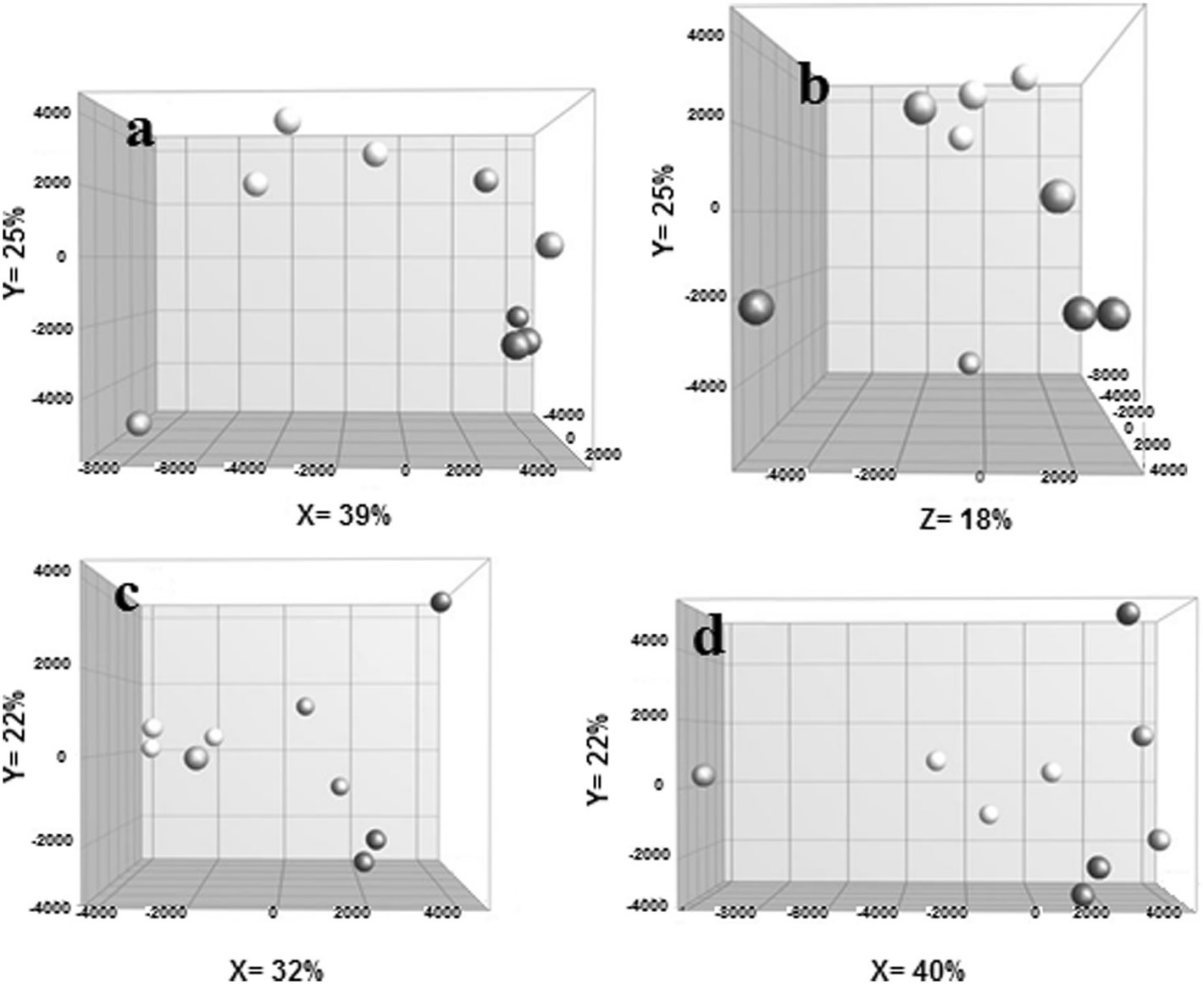
**PCA plots using RMA normalized gene expression data of Col-3 plants and COX + and COX++ lines. a**, PCA plot with PC1 (X) and PC2 (Y). **b**, PCA plot with PC3 (X) and PC2 (Y). **c**, PCA plot using genes with CV < 20% across wild type samples with PC1 (X) and PC2 (Y). **d**, PCA plot using ANOVA insignificant genes (α = 5%) with PC1 (X) and PC2 (Y). Percentages are the variation explained by the corresponding PC. Dark grey circles: wild type samples, light grey: COX + and black: COX++ samples.

A PCA (Principal Component Analysis) plot of the RMA (Robust Multichip Average) normalized data showed distinct clustering of COX + and COX++ lines along PC1, PC2 and PC3 (Figure 4a and b). Col-3 replicates separated from neither of the transgenic lines along these PCs. Clustering of COX + and COX++ lines on different sides of Col-3 suggests a difference in the impact of the two strategies on the transcriptome. The PCA plots also show that the variation in the transcriptome of the transgenic lines along PC1, PC2 and PC3 is within the Col-3 variation, except for variation of COX + lines along PC2 (Figure 4a) and COX++ lines along PC3 (Figure 4b). Col-3 samples showed larger variation along the first PC compared with the transgenic lines (Figure 4a). Also a larger number of highly variable genes were observed in Col-3 than in transgenic replicates as 917 (4.0%), 237 (1.0%) and 242 (1.0%) of the genes had a CV of 20% up to 70% (maximum) in Col-3, to 61% in COX + and to 44% in COX++ , respectively. A PCA after excluding the highly variable genes (with a CV > 20%) shortened the visual distance between the Col-3 replicates (Figure 4c). However, it did not change the overall conformation of the groups relative to each other. To check whether the observed variation within the Col-3 plants of this study had a true biological origin, we performed ORA on the set of genes with CV > 20% in Col-3. The most represented subcategories in the test set belonged to the catalytic activity, primary metabolic process and response to abiotic stimulus categories.To confirm that the observed variation within Col-3 replicates is independent from the variation in differential genes between Col-3, COX + and COX++, all significantly different genes were filtered out by ANOVA (α = 0.05). PCA on the remaining genes showed that groups became closer, but a large variation was still observed within Col-3 replicates (Figure 4d). This observation confirms independence of the large variation within Col-3 replicates from between group variation.

#### ***Transcriptome distance between GM lines and the wild type background***

**Figure 5:**
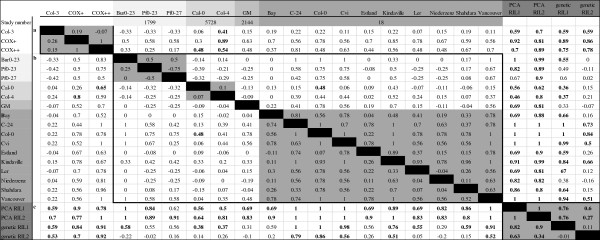
***Transcriptome distances *between *Arabidopsis *genotypes.** Values above the diagonal are the distances based on intact (un-weighted) PC scores and values below the diagonal are distances based on the weighted scores on the first 9 PCs of a PCA on gene expression data of the wild type and transgenic lines of this study **(a)**, wild type accessions of public databases **(b)** and groups of the Cvi/Ler RIL population **(c)**. Significant values are shown in bold (permutation test, P-value = <0.05). a, b and c correspond to the source of the data which is delimited by blocks. Accessions shaded by the same color belong to the same experiment.

PCA can visualize the relationship between samples. However, it is only possible to judge similarity or differences between samples based on three PCs (for our study explaining ~20% of the variation). We used the approach of Houshyani *et al*. (2011), which was used to calculate metabolic hyper-plane distance, to calculate transcriptome hyper-plane distance using the sample scores on the first 9 PCs. For hyper-plane distance calculation, the microarray signals representing *FPS1L* and *HMGR1S* were removed from the dataset. Col-3 and transgenic lines’ sample scores on the first 9 PCs of a PCA with *meta data* was used for calculation of the *transcriptome distance* between Col-3 plants and COX + and COX++ lines (Figure 5a). Using either intact or weighted scores, the Col-3 *transcriptome distance* to COX + (0.19 and 0.26, respectively) and COX++ (-0.07 and 0.15) were not significant (permutation test, P-value = <0.05). The set of independently transformed COX + and COX++ lines showed the maximum possible transcriptome distance to each other (1.0, 1.0), although this was still not significant (Figure 5a). This indicates the absence of any similarity between the COX + and COX++ GM plants in the response to transformation.

### Transcriptome difference assessment

To assess the distances between transgenic lines and Col-3, we evaluated them in the context of natural variation (different accessions) or conventional breeding practices (RILs). For this purpose we used published transcriptome data of different accessions and RILs (the *meta data*) and analysed the differences and distances between accessions and groups of RILs. For analysis the RIL population was divided into two groups (GPs) based on molecular marker differences (Genetic GPs) and based on transcriptional expression differences (Expression GPs). ANOVA on the accessions, Genetic GPs and Expression GPs showed that 137 of 174 and 85 of 98 differentially expressed genes (>2 fold) in COX + and COX++ vs. Col-3, respectively, were also significantly > 2 fold different in at least one of 3 selected experiments from the public database (where statistical analysis was possible) and/or between Genetic GPs and/or Expression GPs. This indicates that a considerable number of differences between the COX + or COX++ lines and the Col-3 also occur naturally and are hence not specific to the genetic modification (for interpretation of the specific changes, see the ORA results section).

Subsequently, the *transcriptome distance* between all groups of the *meta data* was calculated and tested for statistical significance (Figure 5b and c). Within the accessions of the public database, only two accessions (Col-0 in study 18 and Cal-0 in study 5728) had a significant *transcriptome distance* to each other (permutation test, P-value = <0.05) with both intact (0.48) and weighted (0.48) scores (Figure 5b). Although the rest of the pairwise distances were not statistically significant, strong variation was observed, particularly in study number 18. *Transcriptome distances* based on weighted scores in this study were varying from -0.11 between Bay and Estland to 1.00 (the maximum) between several pairs such as Col-0 and Cvi. Using intact scores, Bay and Estland showed a distance of 0.04 which was one of the smallest distances after Niederzenz and Ler (-0.04). Ler is an accession of *Arabidopsis* widely used for both molecular and genetic studies [21]. It was isolated from a mutagenized seed population and harbours the *erecta (er)* mutation that causes strong phenotypes such as altered organ shape, compact inflorescence with flowers clustering at the top and round leaves with short petioles and short and blunt siliques [21]. A look at the Ler *transcriptome distance* by weighted scores and comparing with the rest of the accessions in study 18 revealed a distance range between 0.04 with Niederzenz and 0.78 with Col-0. There are several other pairs of accessions with the maximum distance (1.0) in the same study (such as Cvi and Col-0) (Figure 5b).

The Genetic GPs and Expression GPs comprised RILs that were grouped based on genetic or expression profile similarity, respectively. As every individual of a RIL population represents randomly 50% of each parent’s genome, it is not possible to choose two groups that are genetically most distant. Therefore, AFLP molecular marker data of the RIL population were used in a PCA analysis to select the two genetically most distant groups (Genetic GPs). Two other groups (Expression GPs) were selected on the first three PCs of a PCA plot performed on expression data of the RIL population.

*Transcriptome distances* between Genetic GPs were 0.11 and -0.01 using intact and weighted scores, respectively (Figure 5c). The *transcriptome distance* between Expression GPs was significant and 1.0 (the maximum) for both intact and weighted scores (permutation test, P-value = <0.05) (Figure 5c). There are also significant distances between Genetic GPs and Expression GPs ranging from 0.27 to 0.76 and 0.34 to 0.90 for intact and weighted PCA scores, respectively (permutation test, P-value = <0.05) (Figure 5c).

## Discussion

### Pleiotropic transcriptional effects in the GM plants are smaller than pleiotropic variation in nature

Here we have used different methods (uni-variate statistics, GSEA, ORA, multivariate data analysis and the newly developed tool, hyper-plane distance) to detect, interpret and assess the unintended changes in the transcriptome of transgenic lines compared with Col-3. To assess the global differences, the hyper-plane distance method as previously described [12] was put into context by comparing the *transcriptome distance* between Col-3 and two transgenic lines in Col-3 background (data generated in this study) with the transcriptome distance between different accessions and groups of a RIL population, based on data obtained from public databases. Results show that the largest *transcriptome distances* and statistically significant differences are found between the two groups of the RIL population (Figure 5). This demonstrates that the cross of two different parental lines, resulting in a population of individual offspring plants of mixed genome composition, has a larger pleiotropic effect on gene transcriptional activity than introduction of the two or three (trans)genes in our transgenic lines. Also the global transcriptome differences between the individual accessions of *Arabidopsis* that can be found in nature are larger than the transcriptional differences between the two GM lines and the wild type *Arabidopsis* Col-3.

The difference in transcriptome activity of the genetically diverse *Arabidopsis* accessions represents the genetic variation derived from differences in evolutionary genetic drift between accessions (Figure 2a). This variation still results in very similar phenotypes of the different accessions at a macroscopic level. Presumably, this is due to the multiple levels of feedback regulation that occurs at the transcript, protein and metabolite levels, a phenomenon referred to as phenotypic buffering [22]. In contrast, the macroscopic phenotypic differences between members of the RIL population (Figure 2b) are much larger and can exceed those of the original parental lines. For instance, individual members of two RIL populations (Ler/Cvi and Ler/Col-0) showed extensive variation in clock period and phase, while the parental lines were similar [23]. With the same Cvi/Ler population it was also shown that the balanced gene expression pattern in the parents became transgressive among the segregants of the RIL population as 15% of the genes for which the parents did not show a significant difference in expression levels had an eQTL in the population. Also, much lower heritabilities were calculated from the parental data than with those from the RIL population [24]. Our results on the transcriptional distances are in agreement with results obtained from metabolomics that showed that 40% of the detected masses were specific to the RIL individuals and were not detected in the parental lines [24].

As the variation between accessions and within RILs are considered to be of ‘natural’ origin (in contrast to the genetic modifications in GM plants), the transcriptional distances in that germplasm can be considered as a *baseline*. When the pleiotropic effects on gene expression in GM plants are evaluated in the context of this *baseline*, the results show that the pleiotropic global transcriptome changes in the two GM lines fall well within the range of transcriptome distances that occur in nature. As such, the GM lines may therefore be called substantially equivalent to the naturally occurring *Arabidopsis* accessions.

### Natural variation covers a large part of the transcriptome differences of the transgenic lines with their wild counterparts

Although the quantity of intended and unintended changes in gene expression are minor compared with the number of analyzed genes, there are still many unintended changes in gene activity in the two GM lines. Without any *p-value* adjustment for multiple tests or filtering based on fold change in the expression, 2.4% and 2.1% of the genes were differentially expressed in COX + and COX++ lines, respectively, compared with the wild type. The use of two fold as filter, decreased these numbers to 0.8% and 0.4% for COX + and COX++ lines, respectively. Similar levels of transcriptome changes were observed by introduction of a marker gene to *Arabidopsis *[25] or a sheep serotonin N-acetyltransferase to rice [26].

The specificity of the transcriptional changes was evaluated by GSEA and ORA, which did not result in any consistent change. Moreover, for a considerable number of the genes that showed differences between the GM and WT plants, similar differences were identified in the accessions and RIL population. The fact that a similar difference in gene activity can be found in natural populations does not refer to any specific impact of such difference in gene activity, which could still be a matter of concern in relation to evaluation of GM plants. However, it does show that pleiotropic effects on gene activity occur just as well in nature as well as in classical breeding strategies, even more so than in GM plants.

### Different cause of pleiotropic changes in transcriptome of GM plants

**Figure 6:**
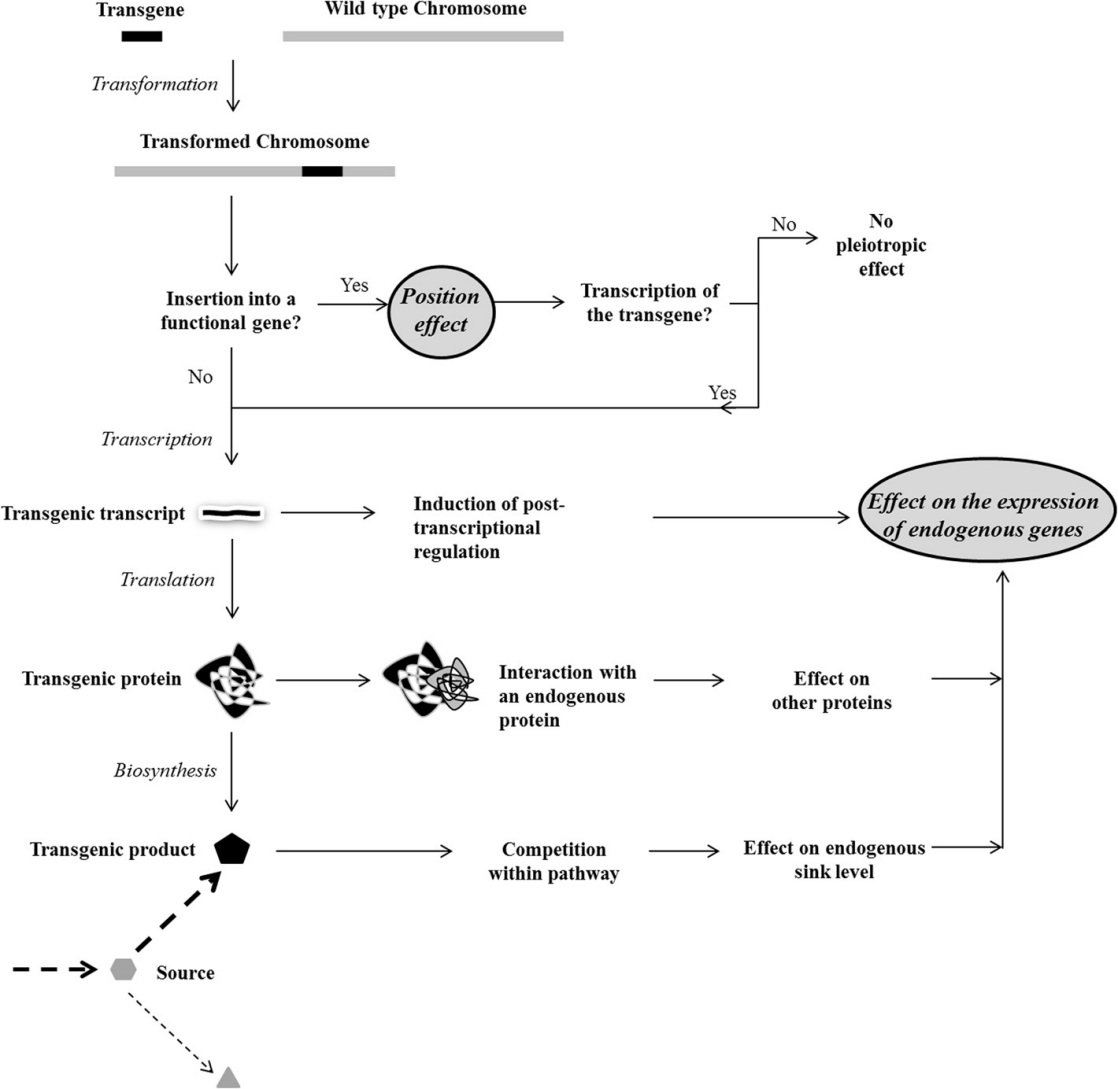
A schematic overview of the possible position and pleiotropic effects of inserted construct and/or gene(s).

Quantitative transcriptome comparison revealed different patterns of up-regulated and down-regulated genes in COX + and COX++, with only three down-regulated genes (>2 fold) in common in both lines. The more global analyses by GSEA or ORA did not yield any commonly changed gene set in COX + and COX++. These findings show that the two strategies that were used to create the same trait (improved biological control of arthropod herbivores) have a different impact on the transcriptome. The pleiotropic changes in the COX + and COX++ transgenic lines seem to be non-specific to the novel trait, as these lines cluster separately from each other (Figure 4). This also suggests a consistent change in the transcriptome of the biological replicates (within each set), which therefore seems to be related to the effect of the introduced gene(s). The deviation from a tight clustering of sets could be the result of additional pleiotropic effects that are independent of the introduced gene(s), and may be the result of pleiotropic effects of different genomic insertion sites (position effect) (Figure 6). Alternatively, deviation from tight clustering of biological replicates can be the result of micro-environmental differences within the experiment, in which case they are not specific to our modification and not reproducible.

### Environmental effects increase baseline variation and further reduce significance of transcriptional distances

Transcription is a polymorphic trait that is under the control of many genetic, epigenetic and environmental factors [27,28] and natural polymorphism among *Arabidopsis* accessions has been already reported to be considerable at the genome [29] as well as transcriptome levels [30]. We note that our *baseline* was constructed using public data of *Arabidopsis* samples that were grown under strict environmental control and harvested at vegetative developmental stages (Additional file 2: Table S1). In real agricultural practice with a higher variability in environmental conditions [12,31], the *baseline* variation would increase and hence the *transcriptome distance* of a genetically modified plant with this baseline would be smaller. Therefore, when environmental effects on transcription would be included, the GM lines likely exhibit even stronger substantial equivalence to natural occurring genotypes than what they show here.

## Conclusions

The current study explored and assessed the changes in the transcriptome of genetically engineered lines of *Arabidopsis* using a holistic *omics* analytical and statistical approach as well as comparison to a natural transcriptome *baseline*. Results show that the pleiotropic changes in the transcriptome of GM plants are non-significant when compared with natural occurring genotypes and that transcriptional distances between GM and untransformed WT are much smaller than the transcriptional distances occurring within the *baseline* group (accessions, RILS). According to the definition by the OECD of “substantial equivalence” [1] this would imply that our transgenic lines should be considered the same as and as safe as conventional *Arabidopsis* lines (the safe counterpart) as they have the same transcriptional characteristics.

## Methods

### Plant material

The generation of COX + and COX++ transgenic lines in *Arabidopsis thaliana* (accession Col-3) background is described elsewhere (Figure 1) [17]. Transgenic lines and wild type plants were grown on LB medium (purified agar 0.8% + 2.2 gr L^-1^ 0.5 MS), supplemented with the herbicide BASTA (10 μg mL^-1^) for transgenic line selection. Plates were placed in a growth chamber (21 ± 2°C with L8:D16 photoperiod with 80–110 μmol.m^-2^.s^-1^ PPF). When seedlings (biological replicates) had 2 true leaves they were transferred to soil (Lentse potgrond, Lent, The Netherlands) and grown for 6 weeks under the same conditions. Growth of COX + and COX++ lines was slightly retarded during the first 4 weeks after transplanting, but this difference disappeared in the last 2 weeks before sampling.

### Total RNA extraction, reverse transcription and qPCR analysis

Sampling on wild type plants and transgenic lines was done by collecting four young fully expanded leaves of a plant (biological replicate). For the transgenic lines sampling was done if insect (*Diadegma semiclausum*) preference for its volatile blend was observed, indicating that the plant was producing the expected volatiles [17]. Therefore sampling was done during a period of five days in the same time of the day and under the same environmental conditions as described above. Samples (biological replicates) were immediately flash frozen in liquid nitrogen, stored at -80°C, ground in liquid nitrogen and total RNA was isolated using the protocol of TriPure Isolation Reagent (Roche Applied Science, http://www.roche-applied-science.com). Total RNA of every biological replicate was treated with DNaseI (Invitrogen, http://www.invitrogen.com) according to the manufacturer’s instructions and purified using the RNeasy Mini Kit (Qiagen, http://www.qiagen.com) and the RNA Cleanup protocol. RNA samples were quantified (UV absorption at 260 nm) using an ND1000 Spectrometer (Nanodrop technologies, http://www.nanodrop.com). OD 260/280 nm absorption ratio (>2) and agarose gel electrophoresis analysis confirmed the purity and integrity of RNA samples.

One μg total RNA of every biological replicate was subsequently converted to cDNA using the iScript cDNA synthesis kit (Bio-Rad, http://www.bio-rad.com). Gene-specific primers were designed using Beacon Designer 7.0 (Premier Biosoft, http://www.premierbiosoft.com) (Additional file 3: Table S2). Primers were checked for gene specificity by blasting against the *Arabidopsis* genome and RefSeq RNA database and by performing melt curve analysis on a MyIQ Single-Color Real-Time PCR Detection System (BioRad). The amplification efficiency of PCR primers was determined by performing RT-PCR on dilution series of a template.

Quantitative RT-PCR analysis was carried out in optical 96-well plates with a MyIQ Single-Color Real-Time PCR Detection System (BioRad), using SYBR Green. Each reaction contained 10 μl 2× iQTM SYBR Green Supermix Reagent (BioRad), 20 ng cDNA and 150 nM of each gene-specific primer in a final volume of 20 μl. All qRT-PCR experiments were performed in duplicate. The following PCR program was used for all PCR analyses: 95°C for 3 min; 40 cycles of 95°C for 10 s and 60°C for 30 s. Threshold cycle (Ct) values were calculated using the MyIQ Optical System software (version 2.0, BioRad). Subsequently, Ct values were normalized for differences in cDNA synthesis by subtracting the Ct value of the reference gene β-tubulin [32] from the Ct value of the gene of interest. Normalized Ct values (δCt) were used for statistical comparison of wild type and transgenic lines using SPSS (t-test, α = 0.05) (IBM, http://www.ibm.com).

### RNA labelling, microarray hybridization and data processing

After qPCR confirmed expression of *FaNES1*, over-expression of *FPS1L* and *HMGR1S* and emission of nerolidol in COX + and COX++ transgenic lines (biological replicates) [17], the total RNA of 3 wild type plants, 3 COX + and 3 COX++ lines (biological replicates) were provided to ServiceXS (http://www.servicexs.com), who performed labelling, hybridization, quality control and data acquisition. Briefly, the RNA concentration and 260/280 nm absorbance ratio measured by the NanoDrop ND-1000 Spectrophotometer (NanoDrop technologies) and electropherograms (plot of results from an electrophoresis analysis) and RNA integrity number produced by the Agilent 2100 Bioanalyzer (Lab-on-a-chip Technology, Agilent, http://www.chem.agilent.com) were used to re-check the RNA quality before labelling. An RNA sample was considered suitable for array hybridization if it had a concentration of 100–500 ng/μl, 260/280 ratio of around 2.0, intact bands on the gel corresponding to 18S and 28S ribosomal RNA subunits and no chromosomal peaks or RNA degradation products (RIN > 5.0) (http://www.chem.agilent.com/RIN/). Hundred ng of RNA was used to synthesize cDNA and Biotin-labelled cRNA using the Affymetrix 3’ IVT-Express labelling Kit (http://www.affymetrix.com). The labelling controls were added to the RNA before labelling. The possibility of very short cRNA formation that can cause a 3’ – 5’ bias and influences the data analysis was ruled out by lab-on-a-chip analysis (Agilent). Fifteen μg cRNA was used for further fragmentation to prevent secondary structure and probe proximity interference and finally 10 μg for the hybridization to the Affymetrix *Arabidopsis* Genome ATH1 Arrays [33]. The GeneChip Hybridization, Wash and Stain Kit (Affymetrix) was used for the hybridization, washing, staining and scanning of the chips. Thirty μl of labelled material was added to 270 μl hybridization cocktail having hybridization controls added. The Affymetrix protocols were strictly followed. Labelling and hybridization controls showed that the processes were reliable.

The Affymetrix Command Console (v1.1) and Expression Console software (v1.1) provided signal estimation and Quality Control (QC) functionality for the GeneChip Expression Arrays. To check quality and applicability of the generated microarray data, the distribution of the log2-transformed intensities was viewed by boxplots and smoothed histograms. They showed no shift in the distribution of the RMA (Robust Multichip Average) normalized data (Additional file 4: Figure S2). To identify outliers relative to the bulk of samples in the data set, each sample was compared to a reference. Since no experimental reference sample was included, it was generated *in-silico* by calculating the median for each gene across all samples. Subsequently, RLE (Relative Log Expression) and NUSE (Normalized Unscaled Standard Errors) plots were generated for RMA normalized data. Samples centered at zero in an RLE plot showing no outlyers. The histogram of the normalized data using the quantile normalization employed in the RMA algorithms also showed no bias for the amplified samples.

### Meta data preparation

The ArrayExpress database for gene expression experiments in EMBL-EBI (http://www.ebi.ac.uk/arrayexpress) was used to query data obtained with Affymetrix ATH1 chips using the following keywords: “*Arabidopsis”* AND “accession OR ecotype”. Five experiments from about 1000 hits were selected that have used the same tissue in a similar developmental stage and of plants grown in almost similar experimental conditions as in our study (Additional file 2: Table S1). The 5 experiments (E-GEOD-5728 and 12676, E-MEXP-1799 and 2144 and E-TABM-18) consisted of CEL files of 64 chips (samples).

The expression data of a Cvi/Ler RIL population (160 lines) were kindly provided by Dr. J. Keurentjes (Wageningen University). For the sake of statistical analysis and hyper-plane distance calculation, 4 groups of RILs were selected from the RIL population each comprising of 5 lines. Two of these groups (GPs) were separated on the first three PCs of a PCA plot performed on just the expression data of the RIL population (expression GPs) and the other two were separated on the first two PCs of a PCA plot which was produced by the molecular marker data of the population (genetic GPs) (https://cbsgdbase.wur.nl/Arabidopsis/demo/marker/markers-index.php).

Expression data of the RIL population, of the wild type plants of this study and of the ecotypes from studies in the public databases were used to generate the *baseline;* addition of the expression data of transgenic lines to *baseline* formed the *meta data* of this study. Systematic biases resulting from different sources of RNA and batches of microarrays in the *meta data* were removed by the “remove effect” function of GeneMaths XT.

### Data analysis

GeneMaths XT (http://www.applied-maths.com) was used for pre-processing of the image data in the CEL files, visualization and PCA. The normalization of all arrays was performed using the Robust Multichip Average (RMA) algorithm with standard settings. All statistical comparisons were performed in PASW statistics 17 (SPSS) using ANOVA in conjunction with Tukey’s test (α = 0.05). Gene set enrichment analysis [18,19] was done using the GSEA desktop application (http://www.broadinstitute.org) to determine whether an *a priori* defined set of genes shows statistically significant difference between wild type plants and COX + or COX++ transgenic lines. A database of 555 gene sets was used for GSEA [20]. Over- and Under-Representation Analysis (ORA) of functional categories [34] in the test set (a set of differentially expressed genes) was done using GeneTrail (http://genetrail.bioinf.uni-sb.de) and a reference set comprising all gene IDs of *Arabidopsis* ATH1 GeneChip. Venn Diagram Generator (http://www.pangloss.com/seidel/Protocols/venn.cgi) was used for producing Venn diagrams to identify overlapping genes in the set of differentially expressed genes. The method of Houshyani *et al. *[12] for hyper-plane distance was used to calculate the *transcriptome distance* between all used groups and genotypes using scores of samples on the first 9 PCs of a PCA plot using the *meta data*. The 9 principle components were selected using Horn’s Parallel Analysis (http://doyenne.com/Software/files/PA_for_PCA_vs_FA.pdf). The *transcriptome distance* calculation was done using non-weighted PC scores, as well as weighted. For the latter, the scores of samples on each PC were multiplied by the variation that was explained by that PC.

The *transcriptome distance* between two groups varies between 1.00 and -1.00 with 1.00 indicating the maximum distance between two groups or no overlap in the location of the group in the hyper-plane, 0 indicating two completely overlapping groups and -1.00 indicating two groups where one group is a child of the other group.

### Availability of supporting data

All microarray data of this paper have been deposited in LabArchives, LLC (http://www.labarchives.com/) under doi:10.6070/H4SN06XQ and accessible via: https://mynotebook.labarchives.com/doi/NTExMTAuOHwzOTMxNi8zOTMxNi9Ob3RlYm9vay8xNDUwMzA4NjU0fDEyOTc0Mi44/10.6070/H4SN06XQ.

## Competing interest

The authors declare that they have no competing interests.

## Authors’ contributions

BH carried out the transcriptomics and data analyses and wrote the manuscript with supervision of SK who gave the manuscript the current structure and incorporated the concept of pleiotropic effects. RB and HB conceived the study, and participated in its design and coordination. All authors read and approved the final manuscript.

## Supplementary Material

Additional file 1: Figure S1 Genes of which the expression correlates (>0.90) with the expression of *FPS1.* Click here for file

Additional file 2: Table S1 Specifications of the *Arabidopsis* samples of which expression data was collected from public databases. Click here for file

Additional file 3: Table S2 Primers used for qRT-PCR. Click here for file

Additional file 4: Figure S2 Boxplots and smoothed histograms for quality check of the microarray data. Click here for file
