# Supplementary material for: Assessment of pleiotropic transcriptome perturbations in Arabidopsis engineered for indirect insect defence
Source: BMC Plant Biol. 2014 Jun 19;14:170. doi: 10.1186/1471-2229-14-170 (PMC4091741; doi:10.1186/1471-2229-14-170)
Supplement: Additional file 1: Figure S1 — Genes of which the expression correlates (>0.90) with the expression of FPS1. [file 1471-2229-14-170-S1.docx]

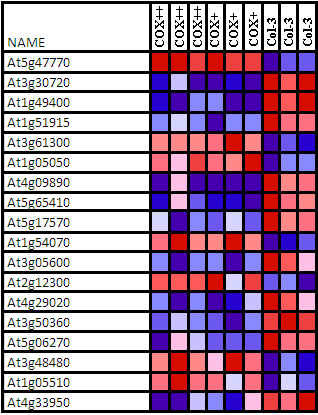


Supplementary Figure 1. Heat map of the gene identities with more than 90% positive or negative correlation with the expression of *FPS1* gene over-expressed in COX+ and COX++ transgenic lines.
