# Supplementary material for: Assessment of pleiotropic transcriptome perturbations in Arabidopsis engineered for indirect insect defence
Source: BMC Plant Biol. 2014 Jun 19;14:170. doi: 10.1186/1471-2229-14-170 (PMC4091741; doi:10.1186/1471-2229-14-170)
Supplement: Additional file 2: Table S1 — Specifications of the Arabidopsis samples of which expression data was collected from public databases. [file 1471-2229-14-170-S2.docx]

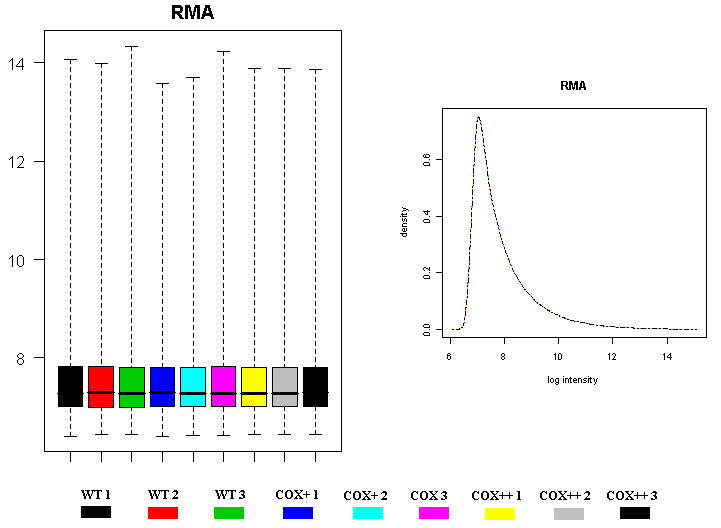


Supplementary Figure 2. Distribution of the log2-transformed intensities after RMA normalization viewed by boxplots and smoothed histograms.
