# Supplementary material for: Assessment of pleiotropic transcriptome perturbations in Arabidopsis engineered for indirect insect defence
Source: BMC Plant Biol. 2014 Jun 19;14:170. doi: 10.1186/1471-2229-14-170 (PMC4091741; doi:10.1186/1471-2229-14-170)
Supplement: Additional file 3: Table S2 — Primers used for qRT-PCR. [file 1471-2229-14-170-S3.docx]

| Supplementary Table 1 . Specifications of the *Arabidopsis* samples of which expression data was collected from public databases. | | | | | | | | |
| --- | --- | --- | --- | --- | --- | --- | --- | --- |
|  | Experiment |  | Tissue |  | Developmental stage during sampling |  | Growth conditions |  |
|  |  |  |  |  |  |  |  |  |
|  | E-GEOD-5728 |  | Leaf |  | Boyes key 3.90^#^ (Rosette growth complete ), 30 days post-sowing |  | 16 hour day lengths on sand watered with 0.5 X Long Ashton solution containing 1 mM CaCl2. |  |
|  | E-GEOD-12676 |  | Leaf |  | 35 days post-sowing |  | 10 hours light (short day) at 20 °C starting from 5 days after sowing |  |
|  | E-MEXP-1799 |  | Leaf |  | 4 weeks after sowing |  | 23 °C, short day on soil |  |
|  | E-MEXP-2144 |  | Leaf |  | 3 weeks after sowing |  | 24 °C, long day on soil |  |
|  | E-TABM-18 |  | Aerial parts |  | 3 weeks after sowing |  | 23 °C, short day on soil |  |
|  |  |  |  |  |  |  |  |  |
| # Boyes D.C. et al.: Growth stage-based phenotypic analysis of Arabidopsis: a model for high throughput functional genomics in plants.The Plant Cell, Vol. 13, 1499–1510, July 2001 | | | | | | | |  |
