# Supplementary material for: Assessment of pleiotropic transcriptome perturbations in Arabidopsis engineered for indirect insect defence
Source: BMC Plant Biol. 2014 Jun 19;14:170. doi: 10.1186/1471-2229-14-170 (PMC4091741; doi:10.1186/1471-2229-14-170)
Supplement: Additional file 4: Figure S2 — Boxplots and smoothed histograms for quality check of the microarray data. [file 1471-2229-14-170-S4.docx]

| Supplementary Table 2. Primers for quantitative RT-PCR analysis | | | |
| --- | --- | --- | --- |
|  | Gene Name | Sequence (5' to 3') |  |
|  | FaNES1 |  |  |
|  | *Fragaria ananassa* | F: CGACTACTGAGACAACATGGTTAC |  |
|  | Nerolidol Synthase 1 | R: TCCTCACCCAGAACTTGCTTG |  |
|  |  |  |  |
|  | FPS1L |  |  |
|  | AT5G47770 | F: ATGAGTGTGAGTTGTTGTTGTAGG |  |
|  | long isoform | R: GCTTTGGATACGACGACGATAG |  |
|  |  |  |  |
|  | HMGR |  |  |
|  | AT1G76490 | F: AAGGGTTACCGTTGGATGGATTTG |  |
|  |  | R: CAAGCAACAATGGACCAGCAATC |  |
|  |  |  |  |
|  | β-Tubulin |  |  |
|  | AtxG | F: CTCAAGAGGTTCTCAGCAGTA |  |
|  |  | R: TCACCTTCTTCATCCGCAGTT |  |
|  |  |  |  |
